# Attenuation of Acute and Chronic Restraint Stress-induced Perturbations in Experimental Animals by *Nelumbo nucifera* Gaertn

**DOI:** 10.4103/0250-474X.42982

**Published:** 2008

**Authors:** M. P. Kulkarni, A. R. Juvekar

**Affiliations:** Department of Pharmaceutical Sciences and Technology, Mumbai University Institute of Chemical Technology, Matunga, Mumbai 400019, India

**Keywords:** *Nelumbo nucifera*, acute stress, cold restraint stress, antistress, adaptogenic

## Abstract

Aqueous extract of leaves of *Nelumbo nucifera* was investigated on acute stress (immobilization stress)-induced biochemical alterations in Swiss mice. The animals were also subjected to acute physical stress (swimming endurance test) and acute chemical stress (writhing test) to gauge the antistress potential of the extract. Further to evaluate the antistress activity of *Nelumbo nucifera* in chronic stress condition, fresh Wistar rats were subjected to cold restraint stress (4° for 1 h) for 7 days after 21 days of pretreatment with the extract. Stimulation of hypothalamus pituitary adrenal axis in stressful condition alters plasma glucose, triglyceride, cholesterol, total protein and corticosterone levels. Pretreatment with the extract significantly ameliorated the stress-induced variations in these biochemical levels in both acute and chronic stress models. The extract treated animals showed increase in swimming endurance time and reduced number of writhes in physical and chemical-induced stress models respectively. Treatment groups also reverted back perturbed neurotransmitter levels (norepinephrine, dopamine and 5-hydroxytryptamine) in brain as well as increase in adrenal gland weights and atrophy of spleen caused by cold chronic stress. In mice immunized with sheep red blood cells, treatment groups subjected to restraint stress prevented the humoral immune response to the antigen. Histopathological studies of adrenal gland of stress control group revealed vacuolar degeneration, loss of architecture and formation of lesions in the cortex, which was reversed by extract treatment. The results indicate that aqueous extract of *Nelumbo nucifera* has significant adaptogenic activity against a variety of biochemical, histological, physiological and immunological perturbations in acute and chronic stress models.

Stress is an aversive stimulus, which perturbs the physiological homeostasis, and its impact is reflected on a variety of biological systems. Complex mechanisms contribute to the breakdown in adaptational processes resulting in various visceral, behavioral and endocrinological changes^1^. It has been postulated that stress is involved in etiopathogenesis of variety of diseases like depression and anxiety, immunosuppression, endocrine disorders, male impotency and cognitive dysfunction to diseases like peptic ulcers, hypertension and ulcerative colitis^2^,^3^. The hypothalamic-pituitary-adrenal (HPA) axis and adrenal glands are particularly crucial for the regulation of stress physiology^1^. More recently involvement of central nervous system (CNS) has been speculated in stress physiology. Suppression of immune competence has been widely reported during stress and complex neuroendocrinal mechanisms play a crucial role in such stress-induced immune modulation. Adaptogens are agents that attenuate/or prevent these effects of stress on the organisms and benzodiazepines such as diazepam are well known for their antistress properties^4^. Many herbs reported in ancient literature have potent antistress activity and their utilities in current scenario need to be unveiled. The indigenous drug *Nelumbo nucifera* (NN) family Nymphaceae (Gaertn.) contains number of alkaloids; roemerine, nuciferine, nornuciferine, nelumboside, anonaine, 5-methoxy-6-hydroxyaporphine, liensinine, isoliensinine, neferine, lotusine, armepavin, liriodenine, asimilobine that have been isolated from leaves, seeds and embryo. It has been reported that nuciferine the major alkaloid isolated from leaves of NN has divergent psychopharmacological activities^5^. Hence it was thought prudent to experimentally evaluate the potential usefulness of leaves of NN for antistress and adaptogenic activity. The aim of the study was to evaluate the effect of aqueous extract (AE) of leaves of NN on acute and chronic stress-induced biochemical, immunological and histological perturbations in animal models.

## MATERIALS AND METHODS

Norepinephrine HCl was obtained from Boehringer Ingelheim BI, Germany while dopamine HCl and 5-hydroxy tryptamine creatinine sulphate were procured from Sigma Chemical Co. USA. The biochemical kits used were obtained from E. Merck India Limited, Mumbai. All other chemicals were of analytical grade and were procured from standard local sources. Sheep red blood cells were obtained from Haffkine Biopharmaceutical Corporation Ltd. Mumbai, India.

### Plant material and extraction:

The leaves of NN were obtained from local sources and were identified and authenticated at the Department of Botany, Ruia College, Mumbai. Dried powdered leaves were defatted with Petroleum ether (60-80°) and successively extracted with water. The extract so obtained was dried under vacuum at 45° and used for further studies.

### Animals:

Male Swiss mice (20-25 g) and male Wistar rats (160-180 g) were used for the study. Animals were housed under standard conditions of animal care, 12/12 h light and dark cycle and fed with standard pelleted diet and water *ad libitum*. All animal experimental protocols have been approved by the Institutional Animal Ethics Committee.

### Swimming endurance test (physical stress)^6^:

Swiss male mice were randomly divided into 4 groups of 6 animals each. Treatment groups were pretreated with test drug extract in two different doses (100 and 200 mg/kg, p.o.) for 21 d by oral route. Control group was treated with 0.2% sodium carboxy methyl cellulose in saline; while positive control group received diazepam (2 mg/kg, ip). Swimming test was carried out on 21^st^ d and immobility time was recorded for 30 min.

### Writhing test (chemical-induced stress)^6^:

Treatment groups and treatment schedule was same as that of swimming endurance test mentioned above. At the end of 21^st^ d; all the animals from treatment and control groups were administered 0.1 ml of 6% glacial acetic acid by intraperitonial route. Number of writhes was observed in all groups.

### Acute restraint stress (ARS)^7^:

Swiss mice were divided randomly into 5 groups, each group containing 6 mice. Group I mice received 0.2% sodium carboxy methyl cellulose in saline; (vehicle control). Group II mice received 0.2% sodium carboxy methyl cellulose in saline and stress; (negative control). Group III mice were treated with diazepam (2 mg/kg, ip) and stress; (positive control). Group IV mice were treated with NNAE 100 mg/kg, p.o. and stress. Group V mice were treated with NNAE 200 mg/kg, p.o. and stress.

After pretreatment with NNAE for 7 d, the fore limbs and hind limbs of mice were tied separately and then together securing them with adhesive tape thereby immobilizing them for 2 h on last day. After the induction of stress for 2 h, blood was collected and plasma glucose, triglyceride, cholesterol and total protein levels were estimated.

### Immunological assay^8^:

Mice were immunized with sheep red blood cell (SRBC, 0.5×10^9^ cells/ml/100 g) on day 0. They were then treated with test drugs for 7 d as above. On 7^th^ d, after initial immunization with SRBC, mice were subjected to restraint stress for 2 h. After induction of stress, blood was collected and serum assayed for hemagglutination (highest dilution giving hemagglutination was taken as antibody titre).

### Chronic cold restrain stress (CCRS):

Treatment groups were similar to acute restraint stress; only that male Wistar rats were used instead of mice in chronic stress study. After 21 d of pretreatment with NNAE, the same treatment was continued for 7 d and all animals except for vehicle control group were concomitantly exposed to cold restraint stress (4° for 1 h) during those 7 d. On the last day, the animals were treated as described earlier, exposed to cold restraint stress and sacrificed. The blood samples were collected in heparinized tubes, centrifuged at 5000 *g* for 10 min to obtain the plasma. The animals were dissected and the organs viz. brain, adrenal glands and spleen were removed and their wet weights were recorded. The brains were immediately frozen in liquid nitrogen and preserved at -70° until analyzed for biochemical parameters. The adrenal glands were preserved in 10% formalin and sent for histopathology to Unique Bio-diagnostics Enterprises, Mumbai.

### Biochemical analysis:

Biochemical tests to estimate plasma corticosterone^9^, glucose^10^, triglyceride^11^, cholesterol^12^ and total protein^13^ were performed using standard procedures reported in the literature. Brain catecholamine levels were determined using the following procedure; the whole brain was homogenized in a mixture of 2 ml of 0.01 N HCl and 30 ml of butanol in a glass teflon homogenizer and the catecholamines were extracted simultaneously by the method of Brownlee and Spriggs^14^. The aqueous phase/acid extract was recovered by centrifugation at 3000 *g* for 5 min and was used for estimation of norepinephrine^15^ (NE), dopamine^16^ (DA) and 5-hydroxytryptamine^17^ (5-HT) by fluorimetric assays.

### Adrenal gland histopathology:

The adrenal glands were preserved in neutral buffered 10% formalin. Microscopic sections of these adrenal glands were prepared and observed for histological changes.

### Statistical analysis:

The results obtained in the present study were expressed as the mean±SD. The numerical results were evaluated by application of one way ANOVA with post Dunnett or Bonferroni test wherever applicable for statistical significance and the level of significance was set at p<0.05.

## RESULTS AND DISCUSSION

Mice pretreated with NNAE at 100 mg/kg and 200 mg/kg showed significantly increased swimming endurance time as well as significantly reduced number of writhes compared to vehicle control group in physical and chemical-induced stress respectively (Table 1). When mice were sensitized with SRBC, the humoral immune response was clearly suppressed in acute restraint stress control group. NNAE-treated groups at 100 mg/kg and 200 mg/kg significantly prevented the anticipatory fall in antibody titres comparable to diazepam control group (Table 1). Pretreatment of animals with NNAE at both doses significantly inhibited stress-induced alterations in plasma glucose, triglycerides, cholesterol and total protein levels in acute restraint stress model (Table 2).

**TABLE 1:** EFFECT OF NNAE ON IMMOBILITY TIME, NUMBER OF WRITHINGS AND ANTIBODY TITRE

Treatment	Mean immobility time (s) [A]	Number of writhings/20 min [B]	Mean antibody titre [C]
Control	551.25±40.86	46.80±5.1	6.9±0.69*
Diazepam 2 mg/kg	212.25±8.66*	-	7.1±0.9*
NN(AE) 100 mg/kg	180.66±25.89*	30.00±3.89*	6.0±0.9*
NN(AE) 200 mg/kg	156.09±23.33*	25.25±4.99*	7.0±0.88*
Stress control	-	-	4.8±0.3

Values are mean±SD, n=6 in each group

* P<0.05, significant as compared to control for [A] and [B] and stress control for [C], statistical test employed is ANOVA followed by Dunnett's test.

**TABLE 2:** EFFECT OF NNAE ON DIFFERENT BIOCHEMICAL PARAMETERS IN PLASMA OF ARS-INDUCED MICE

Treatment	Glucose (mg/dl)	Total proteins (g/dl)	Cholesterol (mg/dl)	Triglyceride (mg/dl)
Control	99.27±10.59	4.56±0.81	69.02±12.06	41.12±9.1
Stress control	150.65±20.99*	8.50±1.67*	140.88±22.06*	52.22±8.4
Diazepam (2 mg/kg)	101.50±20.66**	4.82±0.95**	71.25±10.66**	29.63±5.4**
NNAE (100 mg/kg)	92.63±6.74**	4.97±1.56**	43.51±19.66**	38.35±3.9**
NNAE (200 mg/kg)	83.88±23.39**	5.95±0.78**	34.47 (±7.89)**	37.32±5.9**

Values are mean±SD; n=6 in each group

* P<0.05 significant as compared to control

** P<0.05, significant as compared to stress control, statistical test employed is ANOVA followed by post Bonferroni test

Cold restraint stress resulted in significant increase in adrenal gland weight with concomitant decrease in spleen weight in stress control group, which was significantly reverted by NNAE pretreatment at 100 mg/kg and 200 mg/kg (Table 3). Pretreatment of animals with NNAE at both doses also significantly restored back cold restraint stress induced alterations in plasma corticosterone, glucose, triglyceride, cholesterol and total protein levels (Table 4). Further, cold restraint stress significantly decreased NE and DA levels and increased 5-HT levels in brain. NNAE pretreatment at both doses significantly prevented changes in the neurotransmitter levels in brain (Table 5).

**TABLE 3:** EFFECT OF NNAE ON ADRENAL GLAND AND SPLEEN ORGAN BODY WEIGHT INDICES IN CCRS-INDUCED RATS

Treatment	Adrenal gland weight (mg/100 g)	Spleen gland weight (mg/100 g)
Control	15.00±1.9	267±30.1
Stress Control	35.08±5.96*	150±14.9*
Diazepam 2 mg/kg	15.38±1.5**	202±20.10**
NN(AE) 100 mg/kg	9.95±1.95**	222±9.90**
NN(AE) 200 mg/kg	16.05±0.8**	256±15.56**

Values are mean±SD; n = 6 in each group

* P<0.05 significant as compared to control

** P<0.05, significant as compared to stress control, statistical test employed is ANOVA followed by post Bonferroni test.

**TABLE 4:** EFFECT OF NNAE ON DIFFERENT BIOCHEMICAL PARAMETERS IN PLASMA OF CCRS-INDUCED RATS

Treatment	Corticosterone (μg/100 ml)	Glucose (mg/dl)	Total proteins (g/dl)	Cholesterol (mg/dl)	Triglyceride (mg/dl)
Control	100.4±25.55	96.36±18.92	3.56±0.81	69.02±12.06	41.18±7.93
Stress Control	181.0±29.89*	140.89±20.99*	6.36±0.66*	137.25±46.05*	87.73±8.83*
Diazepam 2 mg/kg	92.10±21.60**	98.99±20.66**	4.86±0.52**	53.03±20.11**	65.05± 8.9**
NNAE 100 mg/kg	51.80±7.67**	98.66±19.55**	4.96±0.61**	88.15±12.66**	87.40±12.84
NNAE 200 mg/kg	53.90± 9.19**	97.99±20.99**	4.98±0.81*	79.19±9.83**	42.86±6.60**

Values are mean±SD; n=6 in each group

* P<0.05 significant as compared to control

** P<0.05, significant as compared to stress control, statistical test employed is ANOVA followed by post Bonferroni test.

**TABLE 5:** EFFECT OF NNAE ON BRAIN NEUROTRANSMITTER LEVELS IN CCRS-INDUCED RATS

Treatment	Brain NA level (μg/g)	Brain DA (μg/g)	Brain 5-HT (μg/g)
Control	28.7±0.50	0.89±0.08	0.52±0.04
Stress control	20.2±0.64*	0.49±0.14*	0.75±0.09*
Diazepam 2 mg/kg	36.7±0.80**	0.98±0.11**	0.59±0.06**
NNAE 100 mg/kg	35.3±0.86**	1.09 ± 0.19**	0.52±0.07**
NNAE 200 mg/kg	33.3±0.89**	1.19 ± 0.15**	0.49±0.09**

Values are mean±SD; n=6 in each group

* P<0.05 significant as compared to control

** P<0.05, significant as compared to stress control, statistical test employed is ANOVA followed by post Bonferroni test.

**Fig. 1:**
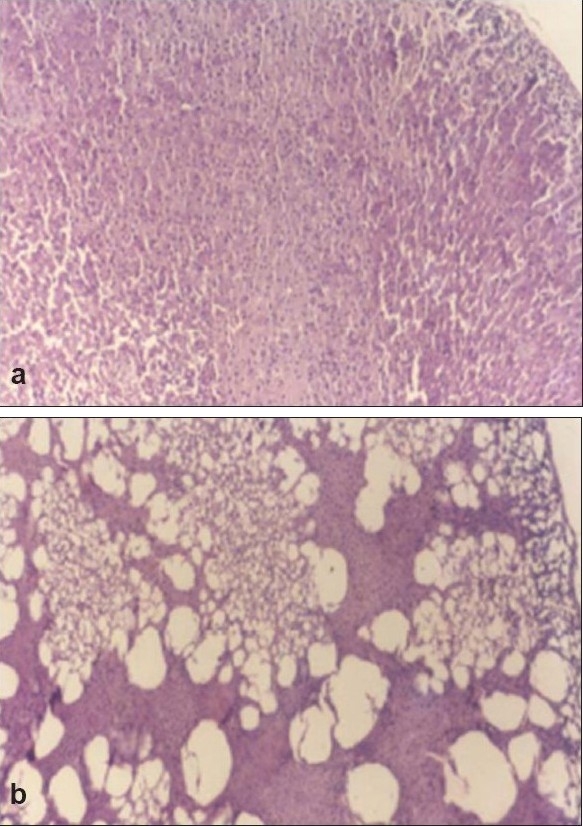
Histopathology sections of adrenal gland of vehicle control and stress control group 1a shows adrenal gland section of vehicle control animal showing normal histology. 1b shows adrenal gland section of stress control animal showing distortion of the cords, loss of architecture, swelling and lipid depletion in cortical region. Magnification was set at 200X and the method of staining was hematoxylin and eosin.

**Fig. 2:**
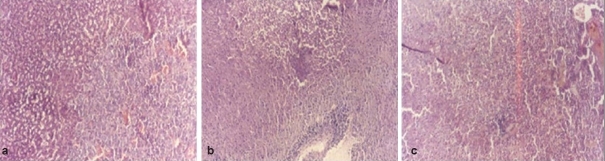
Histopathology sections of adrenal gland of diazepam- and NNAE-treated groups 2a. shows adrenal gland section of diazepam-treated animal. 2b shows the effect on NNAE treated (100 mg/kg, p.o.) adrenal gland section. 2c shows the effect on NNAE-treated (200 mg/kg, p.o.) adrenal gland section. Fig. 2b and 2c clearly show minimal necrosis, swelling and fatty degeneration in adrenal cortex. Magnification was set at 200X and the method of staining was hematoxylin and eosin.

Histopathological profile of normal rat adrenal gland showed regular compact arrangement of cells (fig. 1a), while stress treated animals showed distortion of the cords, loss of architecture, swelling and lipid depletion in cortical region (fig. 1b). Zona fasciculata and zona reticularis had large to small vacuolations and medulla revealed the zones of coalesced empty foci (fig. 1b). Pretreatment with diazepam (fig. 2a) and NNAE at 100 mg/kg and 200 mg/kg, p.o. (fig. 2b and 2c) caused increase in depleted lipid content in adrenal cortex with reduction in large vacuolations in zona fasciculata and zona reticularis. Further, distortion of the cords, loss of architecture and swelling in cortical region was significantly reduced in treatment groups.

The last 20 years of research has taught us much about how the response to stress works. The two main systems involved in stress response are the HPA axis and the sympathetic nervous system. Triggered primarily by an area in the brain stem (lowest part of brain) called the locus coeruleus, the sympathetic nervous system secretes catecholamines. The hypothalamus is a major integrating center for receiving messages from divergent centers and converting them to hormonal signals, via the control of the pituitary gland and by neural pathways^18^. The activation of this HPA system results in secretion of corticotrophin hormone, adrenocorticotropin hormone (ACT), β-endorphin and glucocorticoids into the circulation. Release of ACT in stress stimulates adrenals to increase production of hormones-epinephrine, norepinephrine and corticosteroids^19^. These hormones have profound effect on metabolic functions. Increased plasma cortisol influences the mobilisation of stored fat and carbohydrate reserves^20^, which in turn increase blood glucose, total protein cholesterol and triglyceride levels.

Increased swimming endurance in mice, pretreated with adoptogens has been reported and the test system is used to evaluate the agents with adoptogenic properties^21^. Hence swimming endurance time and number of writhes were used as antistress parameters for preliminary adaptogenic activity screening of the extract. Mice pretreated with NNAE showed significant improvement in the swimming time and reduced number of writhes in acute stress model. Further CNS is an important mediator of immune regulation. Reports have indicated that intervention at the level of the hypothalamus modulates some aspects of immune function. The hypothalamus is one of the neural substrates for the regulation of stress responses and immune function is hampered during such aversive stimuli^8^. In the immunological assay, NNAE pretreatment reversed the restraint stress-induced suppression in the antiSRBC antibody titre, thereby showing its protective action on humoral system.

The increased requirement of adrenal cortical hormones during stress may be one of the reasons for increased adrenal weights in stress control group. NNAE pretreatment resulted in reversion of increase in adrenal gland weight caused due to the stress besides preventing spleen atrophy thus inhibiting the basic signs of stress response. Pretreatment with NNAE have also found to inhibit stress-induced rise in biochemical parameters in plasma in both acute and chronic stress-induced models. The effect observed may be attributed to their protective effect on adrenal glands as shown by the histopathological sections of treatment groups. Further CNS changes due to excessive stress include depleted NE and DA levels along with increase in 5-HT levels in brain. NNAE pretreatment restored back the perturbed neurotransmitter levels in brain, thereby reinforcing its antistress potential.

In conclusion, the present investigation indicates that NNAE has significant adaptogenic activity as shown by its mitigating effects on several acute and chronic stress-induced biochemical, immunological and histological perturbations, comparable to that of diazepam, the conventional antistress agent.
